# Trends In Community and State Rates of HIV/AIDS Among Adults 65+ In New England

**DOI:** 10.1093/geroni/igaf122.2682

**Published:** 2025-12-31

**Authors:** Lien Quach, Taylor Jansen, Elizabeth Dugan

**Affiliations:** University of Massachusetts Boston, Boston, Massachusetts, United States; University of Massachusetts Boston, Boston, Massachusetts, United States; University of Massachusetts Boston, Boston, Massachusetts, United States

## Abstract

HIV/AIDS is a significant public health concern in the U.S., particularly among aging populations. While New England has lower overall HIV/AIDS incidence, disparities persist among racial and ethnic minority group members, LGBTQ+ individuals, and those affected by the opioid epidemic. This study calculates community and state HIV/AIDS prevalence trends among adults aged 65+ across Massachusetts (MA), Connecticut (CT), Rhode Island (RI), New Hampshire (NH), Vermont (VT), and Maine (ME). Data sources included: the Centers for Medicare & Medicaid Services (2012-2021), the American Community Survey (2018-2022), and the CDC’s Behavioral Risk Factor Surveillance System (2010-2022) (see healthyagingdatareports.org). Small area estimation techniques were used to calculate age and sex adjusted rates. ArcGIS was used to map community rates across the region. Results found a steady increase in HIV prevalence across all six states from 2014-2015 to 2020-2021. In 2014-2015, MA (0.17%) and CT (0.18%) had the highest prevalence, while NH (0.05%) and VT (0.08%) were lower. By 2020-2021, the increase in older adults living with HIV/AIDS was most significant in MA (+0.13%) and CT (+0.1%), while ME (+0.08%), NH (+0.05%), and VT (+0.05%) experienced slower growth. These findings highlight geographic disparities in HIV/AIDS prevalence among older adults, with MA and CT showing the fastest increases. State-level factors such as healthcare access, socioeconomic conditions, and policies may contribute. New England’s healthcare infrastructure offers a model for policy-driven prevention. Further research is needed to inform targeted public health strategies and improve outcomes for aging populations infected with HIV/AIDS.

